# Mechanical Stimulation in the Residual Femur During Gait in Transfemoral Prosthesis Users Provides a Potential Reason for Bone Mineral Density Loss

**DOI:** 10.1002/cnm.70103

**Published:** 2025-10-09

**Authors:** Jose L. Zavaleta‐Ruiz, Stefania Fatone, Matthew J. Major, Pankaj Pankaj

**Affiliations:** ^1^ Institute for Bioengineering, School of Engineering The University of Edinburgh Edinburgh UK; ^2^ Department of Rehabilitation Medicine, Division of Prosthetics & Orthotics University of Washington Seattle Washington USA; ^3^ Department of Physical Medicine & Rehabilitation, Feinberg School of Medicine Department of Biomedical Engineering, McCormick School of Engineering Northwestern University Chicago Illinois USA; ^4^ Jesse Brown VA Medical Center Chicago Illinois USA

**Keywords:** finite element analysis, osteopenia, prosthetic socket, transfemoral amputation

## Abstract

Individuals with transfemoral amputation (TFA) experience bone loss in their residual femur at levels seen in bedridden and post‐menopausal individuals. It has been suggested that the time until first prosthesis fitting, gait deviations, and muscle atrophy may be contributing factors, but evidence is inconsistent. Prosthetic sockets are typically designed to off‐load the distal end of the residual limb, yet the effect of the load transmission pathways of the prosthetic socket on a residual femur has not been examined. Using existing datasets, we recreated the prosthetic socket environment within finite element (FE) models by extracting the skeletal geometries of 10 able‐bodied individuals from computer tomography scans, and anthropometrically pairing them with gait data acquired from individuals with unilateral TFA. Normal skeletal geometries were modified to resemble a TFA and fit with an ischial containment socket (ICS). The modified skeleton was positioned with respect to the socket using motion analysis marker locations and tested using the ground reaction forces corresponding to three gait instances from at least four steps. Additional mirror models without the ICS were created for comparison. We validated our study by comparing hip forces from the original gait data to acetabular contact forces estimated using the FE models. We found that the residual femur wearing an ICS experienced mean compressive strain of −105 ± 42 μE and −722 ± 155 μE without the ICS. Simulations show that this is because the ICS redirects load through the pelvis, diminishing force transmission from the femoral head to the acetabulum.

## Graphical abstract

Otherwise healthy individuals with transfemoral amputation, even young athletes and servicemen, are consistently diagnosed with low levels of bone mineral density in their residual limb. Our computational analysis examined the effect of a prosthetic socket on the mechanical stimulus in 10 subjects under three fittings during ground reaction forces of their recorded gait. Our results show that pelvic reliance of the socket, an interaction by design of the prosthetic device, reduces the compressive strain stimulus to below a threshold known as “disuse window” throughout the gait cycle.

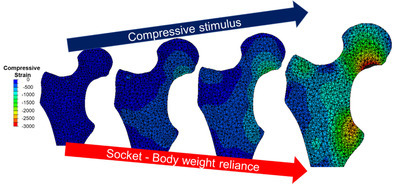

## 1 Introduction

Studies dating back to 1969 [1] report that, after transfemoral amputation (TFA), the residual femur experiences bone demineralization at bone mineral density (BMD) levels consistent with osteopenia in up to 90% of subject samples [2, 3, 4, 5, 6]. These studies also report relatively healthy BMD levels in amputations distal to the femur (e.g., knee disarticulation and transtibial amputation) [7]. However, there remains no consensus regarding the mechanisms underlying BMD loss in the TFA population, with most explanations related to a diminished mechanical stimulus either by low mobility levels [8, 9] or due to gait alterations inherent to both the severance of muscles by amputation [10, 11] and limitations in prosthetic alterations of normal joint kinematics and kinetics [3, 9, 12].

Unfortunately, early rehabilitation aimed at increasing physical activity and shortening bedrest after surgery has not been successful at minimising progressive BMD loss secondary to amputation [8]. While there is some suggestion that the prosthesis itself may share responsibility for BMD loss [8, 12, 13, 14], the influence of its pelvic reliance on the altered loading mechanism has not been investigated. Moreover, while technological advancements in prosthesis design have allowed individuals with high mobility to participate in impact activities [15], which are known to result in improvements in BMD [16], the evidence, however, shows that this has not happened [3, 7] for individuals with TFA. Although socket design is rarely reported, the predominantly used design since the 1980s has been the ischial containment socket (ICS) [17, 18]. The ICS is designed to utilise the ischial tuberosity of the pelvis and gluteal muscles as the main source of load‐bearing support, transferring ground forces through the prosthesis while bypassing the residual femur [19], with the intention of limiting the load experienced by the sensitive distal end of the amputated femur and hence enhancing comfort and minimising the risk of tissue trauma. This history of low BMD in transfemoral prosthesis users led us to consider the protective influence of the ICS on distal femur loading in relation to the trajectorial theory as per Wolff's law [20], along with the Minimally Effective Strain for bone remodelling described in the mechanostat theory [21, 22]. The mechanostat theory proposes that if mechanical stimulation above a threshold strain (typically 300 μE) is not attained, then osteoclast activity is overstimulated and bone resorption activated [23].

A recent proof‐of‐concept computational study [24] showed the constant influence of pelvic loading reliance in the prosthetic socket on the mechanical stimulus imparted to the residual femur at four hip positions. This study presents finite element (FE) analyses using gait data from individuals with TFA at three gait instances with the aim of evaluating the capacity of the ICS design to modify the mechanical environment created in the residual femur and relating it to the Mechanostat adaptation theory [21] to assess whether it offers an explanation for bone mineral density loss.

## 2 Methods

### 2.1 Data Sources

FE models were constructed using two existing data sources: an open access repository of computed tomography (CT) scans at https://weisslabutah.org/ [25] and gait data from authors of a previously conducted clinical trial [26]. Due to the use of existing data, no institutional review board or ethics committee approval was required for this study.

CT scans from 10 healthy individuals (5 females, 5 males; age, 26.4 ± 3.9 years; height, 174 ± 6.5 cm; weight, 70 ± 12.6 kg; body mass index [BMI], 22.9 ± 3.5) were sourced from an open access repository [25] and matched by sex, weight, and height with previously collected gait data [26] from seven individuals with unilateral TFA and high mobility levels wearing an ICS (3 females, 4 males; age, 40.3 ± 12.5 years; height, 173 ± 12.4 cm; weight, 73.9 ± 16.5 kg; BMI, 24.4 ± 3.8). Individual CT scans were paired with data from TFA participants using BMI (±five units). Data from three TFA participants were paired with six CT scan participants to ensure BMI matching, as described in Table 1. Sex was used for skeletal geometry matching. Age and other categorical variables related to tissue material properties were not considered relevant as models explored the homogeneity of ICS effect.

**TABLE 1:** Characteristics for matched pairs of subjects.

Subjects			Differences between paired subjects^a^				Residual limb length (cm)^c^
CT^b^	ICS^c^	Sex	Age	Height (cm)	Mass (kg)	BMI	
1	9	Female	2	6	−1	−2.1	22.8
2	6	Male	−18	−7	1.2	1.9	22.2
3	25	Female	3	12.5	−4.6	−5	18
4	4	Male	−32	−3.5	0.2	0.8	20
5	24	Female	−38	3.5	−6.9	−3.1	26
6	24	Female	−38	−0.5	−13.9	−4.5	26
7	2	Male	−7	−2.5	−11.6	−2.8	24.3
8	4	Male	−24	−3.5	4.2	2.1	20
9	26	Male	−2	0	−5.6	−1.7	25.5
10	9	Female	−8	7	19	4.9	22.8

Abbreviations: BMI, body mass index; CT, computer tomography group; ICS, ischial containment socket group.

^a^ Subject indicated difference as CT minus ICS.

^b^ Subject identifier corresponds to data from Harris et al. [25].

^c^ Subject identifier corresponds to data from Fatone et al. [27].

The gait data used in FE modelling were collected from participants with TFA as they walked back and forth across a 10 m walkway using a 12‐camera optical motion capture system and six force platforms embedded in a 10‐m level walkway (see Fatone et al. [26] for details). Ground reaction forces for the FE models were obtained from gait data spanning at least four steps (heel contact to toe‐off) recorded on a single force platform.

### 2.2 Preparation of Model Geometries

Pelvic and femoral bones were extracted from CT scans using Simpleware ScanIP (Synopsys Inc., Sunnyvale, CA, USA), segmented into cortical and trabecular regions, and manipulated in SolidWorks (Dassault Systèmes, Vélizy‐Villacoublay, France) to match the residual limb length reported for individuals in the TFA sample (Table 1). The space between the acetabulum and the femoral head was also extracted from CT scans and modelled as a capsule representing a bridge between the two geometries. An ICS was fitted to the bone geometries, positioning the ischial tuberosity within a posterior, inferior, and medial ischial containment wall, cupping the greater trochanter with the socket's lateral wall, and locating the socket's adductor longus relief in the medial anterior corner [28]. Individual homogeneous soft tissue mass, as final geometry, was reconstructed matching the inside volume of the corresponding socket. Musculoskeletal and ICS geometries were then exported into the FE package ABAQUS (Dassault Systèmes, Vélizy‐Villacoublay, France).

### 2.3 Testing Environment

**FIGURE 1:**
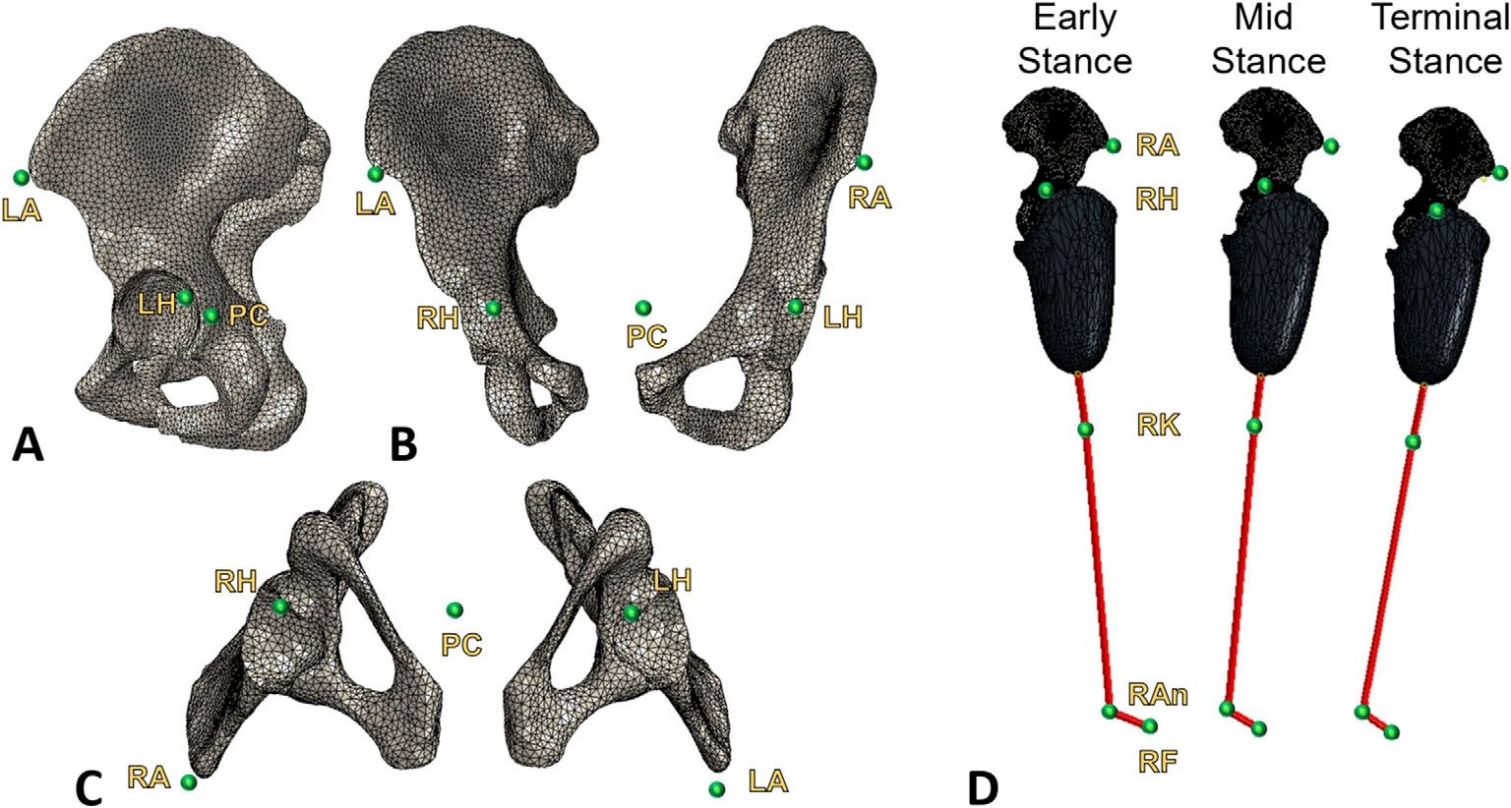
Marker locations as derived from gait data [26]: LA = Left anterior superior iliac spine, RA = Right anterior superior iliac spine. LH = Left hip centre, RH = Right hip centre, PC = Pelvic centre, RK = Right knee centre, RAn = Right ankle centre, and RF = Right Foot centre. Comparison of marker locations with pelvic landmarks in three planes (A) sagittal, (B) coronal, and (C) transverse (inferior view). (D) Modelled skeletal and prosthetic orientation with respect to marker positions during three gait instances.

Three gait instances corresponding to each peak and valley of the vertical ground reaction force (GRF) (referred to as early stance [ES], mid stance [MS], and terminal stance [TS]) were selected for each step. Then the three‐dimensional coordinate position of each individual's pelvis centre of gravity, hip joint centres, left and right ASIS, foot centre of gravity, and joint centre of rotation from both the prosthetic side knee and ankle, as calculated by OrthoTrak (Motion Analysis Corp., Rohnert Park, CA, USA), at these gait instances were plotted in ABAQUS. Importing the marker coordinate system into the FE environment allowed transference of the lab‐based reference frame used during gait analysis, which includes the three‐dimensional (anterior–posterior, medial‐lateral, and vertical) location of the video coordinate system and the top surface of the force plate. Further, the walking direction was used to match the orthogonal hip forces. The positional relationships between each marker and the corresponding pelvic bony landmark are displayed in Figure 1.

The position of the models at each gait instance was performed by matching musculoskeletal tissue and prosthesis to the corresponding marker's coordinate location. The long axis of the femur was aligned towards the knee joint centre marker defining the hip joint orientation. Prosthetic componentry distal to the socket was modelled with beams connecting the socket apex to the knee joint centre. The pylon connecting the prosthetic knee centre to the prosthetic ankle centre was modelled with another beam, and a third beam connecting the ankle centre and foot centre of gravity represented the prosthetic foot as seen in Figure 1D. After anthropometric pairing at ES, MS, and TS instances, proximal coordinate positions for the ASISs, pelvic and hip centre measured with respect to the CT pelvic bone landmarks had a maximum separation of 5 mm.

Finally, the containment was modelled using axial connectors, with a total elasticity equal to the Young's modulus of soft tissue (*E*_con_ = 0.2 MPa), from the ischial tuberosity to the ischial containment area. All FE models were tested under three compressive configurations describing clinicians' ability to create an interaction between the ischium and ramus with the socket and account for subjects' soft tissue compressibility, by including connectors with displacement allowances of 0 mm, 3 mm, and 5 mm (high containment, medium containment, and low containment, respectively) [24, 29].

### 2.4 Boundary Conditions

The model's static evaluation of forces at each gait instance was performed with ABAQUS implicit solver. The location of the pelvic centre was fully restrained via four nodes at the anterior sacroiliac ligaments and another four nodes at the pubic symphysis using a continuum coupling towards the pelvic centre. The following interactions were defined as tied (bonded): the outside surface of the femur trabecular bone to the inside surface of the femur cortical bone, the outside surface of the femur cortical bone to the soft tissue contacting area, the soft tissue perimeter to the inside surface of the ICS, the socket apex node to the thigh pylon proximal node and intersecting nodes from distal prosthetic beams. Finally, at the foot centre node, the three directional ground reaction forces, as captured by the force platforms, were imposed for the analysis of the mechanical stimulus experienced by the femoral trabecular region and quantified as compressive strain.

### 2.5 Mirror Models

Accompanying mirror models, in which there was no socket and in which the distal prosthetic components were directly connected to the residual femur, were also created to match marker positions as in the models with the sockets. In these mirror models, all boundary conditions not related to the socket were retained. These models served as the baseline comparison for the models with the socket.

### 2.6 Material Properties and Mesh

Musculoskeletal and ICS geometries were defined as isotropic and homogeneous with cortical Young's modulus, *E*_c_ = 15 GPa, and Poisson's ratio, ν_*c*_ = 0.3, carbon fibre ICS, *E*_*s*_ = 1.5 GPa, ν_*s*_ = 0.3, trabecular bone *E*_*t*_ = 150 MPa, ν_*t*_ = 0.3, and soft tissue *E*_*st*_ = 0.2 MPa and ν_*st*_ = 0.475 [30, 31]. The capsule between the femoral head and the acetabulum was modelled using isotropic linear elasticity for simplicity with *E*_*cp*_ = 1.5 MPa and ν_*cp*_ = 0.45 [32]. Distal prosthetic componentry was modelled as Timoshenko quadratic beams. Skeletal and soft tissue were represented with 10‐node tetrahedral elements with ~2.25 mm edge length, while beam elements were represented by one element per geometry as bending in the distal prosthetics was not the focus of the study. Mesh convergence studies showed that the average minimum principal strain in the key regions of the femur only deviated by 8.25 μE with a five‐fold increase in element numbers.

### 2.7 Statistical Analysis

For model validation, independent t‐tests were performed to compare hip joint force vector magnitudes estimated using inverse dynamics, by OrthoTrak, during gait to total force due to contact pressure in the acetabular region in FE mirror models as a response to leg position and ground reaction force at each gait instance. Mirror models were used for this comparison as hip force estimations from OrthoTrak did not incorporate bony pelvic containment provided by the ICS. Forces were normalised using body mass and height and averaged for all steps and compared over all three gait instances considered in the three orthogonal directions: anterior to posterior, medial to lateral, and vertical.

Repeated pair measures (i.e., inter‐subject) one‐way ANOVA with Tukey's comparison test was used to compare normalised contact forces registered in the hip joint for each of the three socket pelvic containment conditions (0, 3, and 5 mm) and the mirror models at each gait instance, that is, ES, MS, and TS. A paired t‐test was used to compare the minimum compressive strain between the ICS (0 mm connector condition) and no‐ICS (mirror model) models to assess the effect of the ICS containment for each gait instance. For all analyses, a critical *α* of 0.05 was used (Origin 2023b [Academic], OriginLab Corporation, Northampton, MA, USA).

## 3 Results

In total 258 FE models (129 with socket and 129 mirror) were created for this study, distributed as 10 skeletal geometries, analysed for 4 (or 5) steps and at 3 gait instances.

### 3.1 Validation Using Mirror Models

**FIGURE 2:**
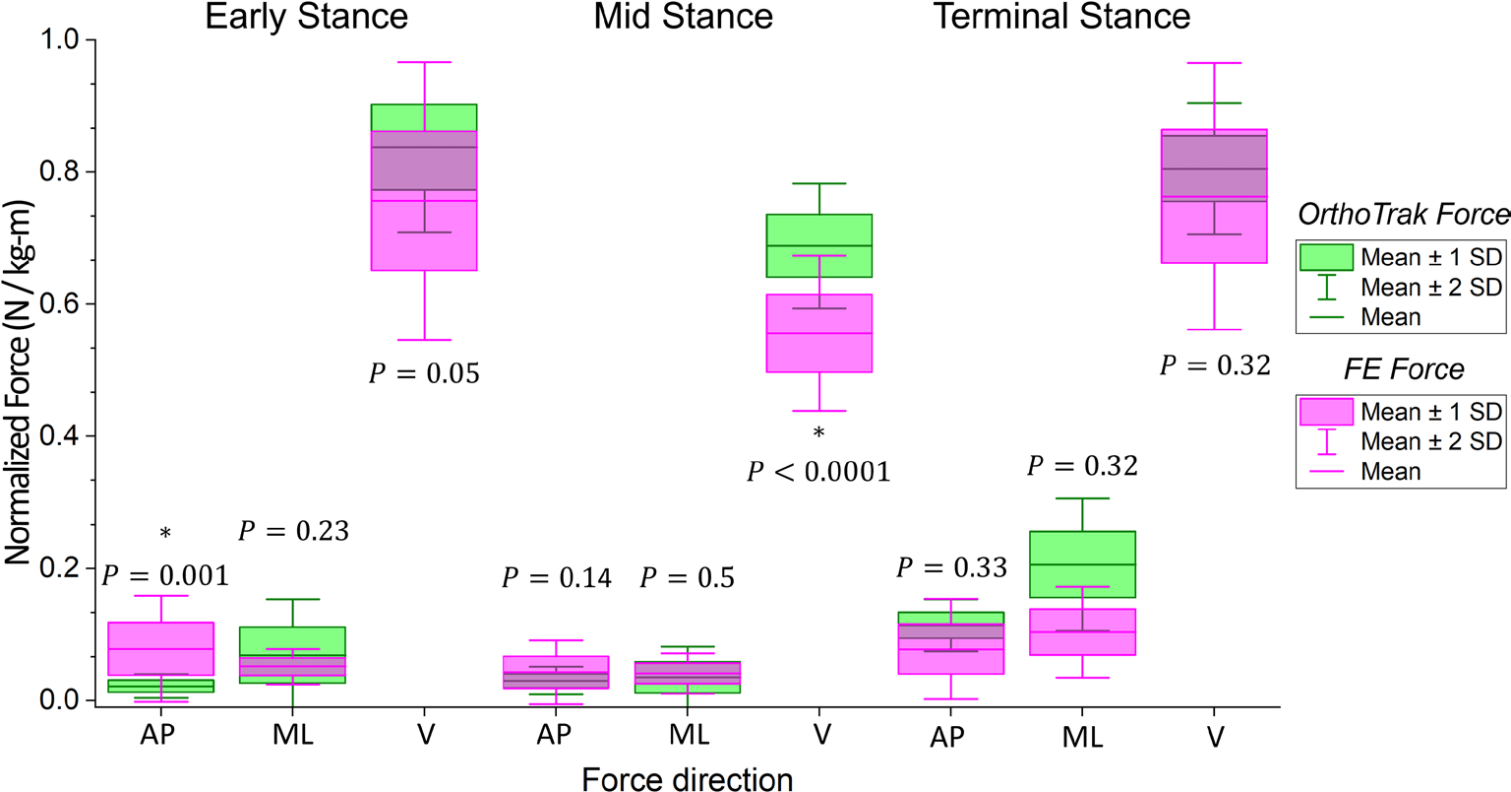
Subjects' hip joint force, normalised using body mass and height. Green boxes are values obtained from trials conducted by Fatone et al. [26] and purple boxes are values from finite element (FE) analyses of mirror models (with pelvic reliance excluded), during early stance, mid stance, and terminal stance. AP = antero‐posterior, ML = medio‐lateral, V = vertical.

Validation results showed no significant difference between force vector magnitudes computed from the FE mirror models (without ICS) and OrthoTrak gait system estimates [26] during ES (*p* = 0.94), MS (*p* = 0.72), and TS (*p* = 0.55), as shown in Table 2. However, analysis of each force component showed a mean significant difference only when comparing the anterior–posterior component at ES (*p* = 0.001) and vertical component at MS (*p* < 0.0001), as shown in Figure 2.

**TABLE 2:** Independent *t‐*tests of normalised force values obtained by OrthoTrak and FE computations.

Gait instance	Model	*N*	Mean	SD	SEM	Median	*t*‐Statistic	Prob > |*t*|
Early stance	OrthoTrak	30	0.298	0.377	0.069	0.08	0.0752	0.94
	FE	30	0.291	0.333	0.061	0.095		
Mid stance	OrthoTrak	30	0.239	0.311	0.057	0.037	0.3599	0.72
	FE	30	0.213	0.249	0.045	0.050		
Terminal stance	OrthoTrak	30	0.309	0.334	0.061	0.126	0.5941	0.55
	FE	30	0.260	0.299	0.055	0.077		

Abbreviations: *N*, samples; SD, standard deviation; SEM, standard error of the mean.

### 3.2 Mechanical Environment With ICS

**FIGURE 3:**
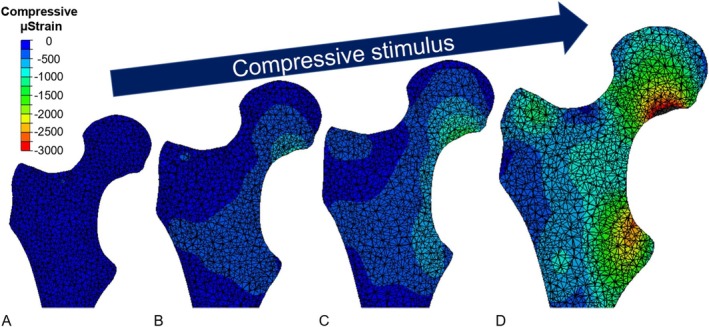
Proximal trabecular bone compressive strain contours during early stance, tested under matching kinematics and ground reaction forces, with (A) high, (B) medium, and (C) low amounts of bony pelvic containment, and (D) without the ischial containment socket (ICS).

**FIGURE 4:**
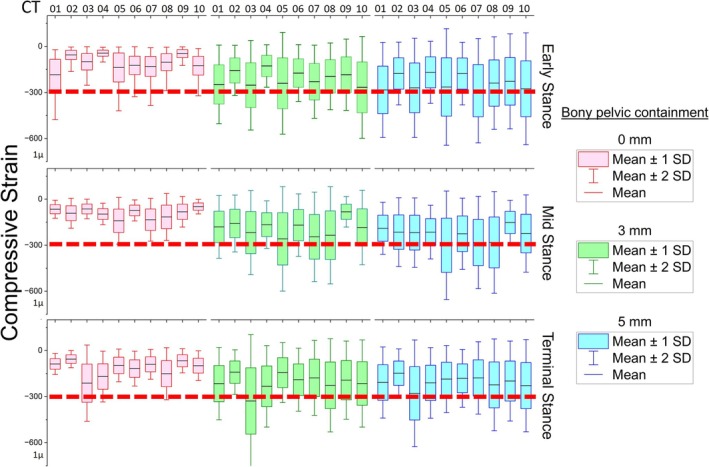
Subjects' compressive strain values during three gait instances (early stance, mid stance and terminal stance) for different amounts of bony pelvic containment (high [0 mm], medium [3 mm], and low [5 mm]). Red dashed line describes the bone remodelling threshold [23].

**FIGURE 5:**
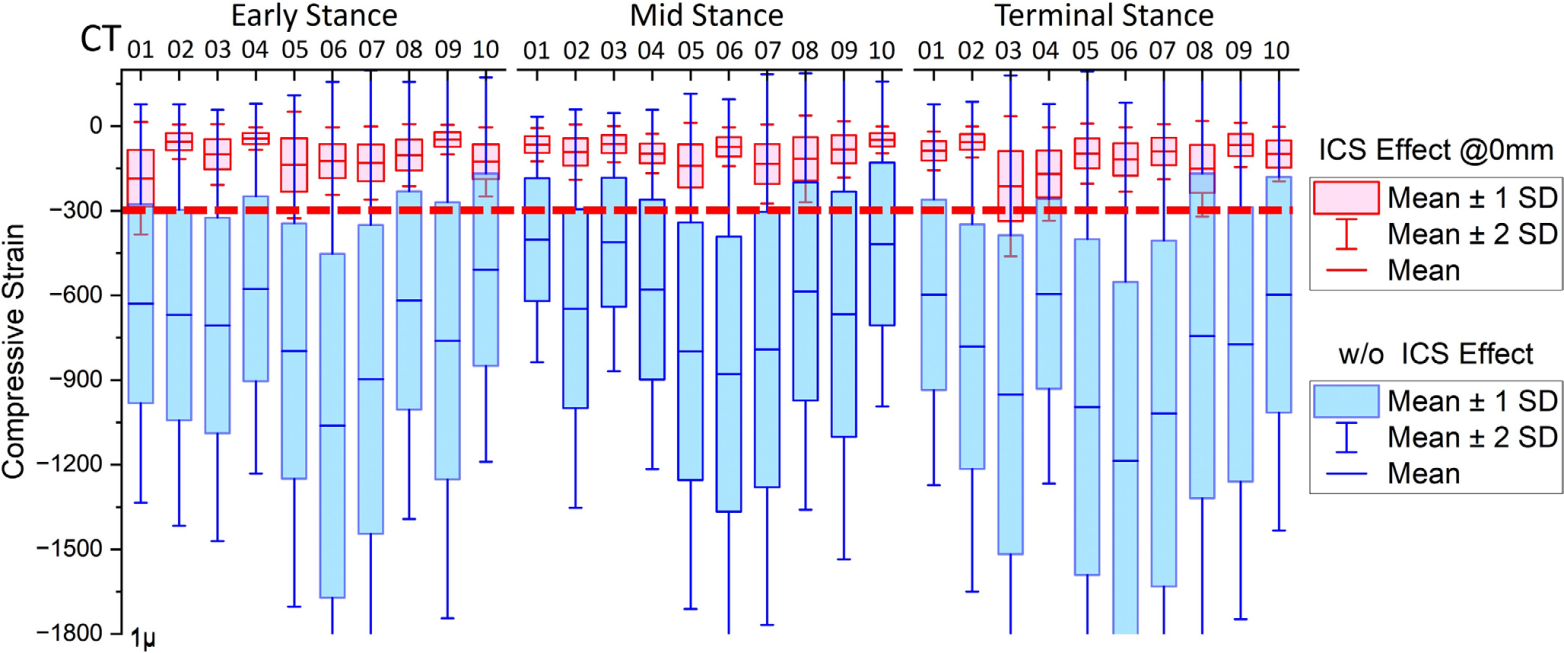
Compressive strain comparison during early stance, mid stance, and terminal stance instances, with high pelvic bony containment of 0 mm separation (red boxes) and without ischial containment socket (ICS) (blue boxes). Red dashed line describes the bone remodelling threshold [23].

Simulations conducted with the ICS showed mean compressive strain patterns registered in the trabecular bone increased as the amount of bony pelvic containment was reduced, as shown in Figure 3 (example in ES). The results across the three gait instances show that 95% of the normalised GRF input is transmitted via the interaction between the ischium and ramus containment within the socket for the high containment scenario, which goes down to 62% when the containment is low. The largest mechanical stimulus, however, was achieved in the absence of the ICS (Figure 3D example in ES). This trend is reflected in the compressive strain values computed for three gait instances (Figure 4) and as previously shown in a proof‐of‐concept study which included a sensitivity analysis [24]. With the greatest amount of bony pelvic containment (0 mm of separation), compressive strain in the residual femur was found to have significantly suppressed mean strain compared to those obtained without the ICS (*p* < 0.0001 across all subjects and gait instances) (Figure 5).

Femoral trabecular compressive strain averages for all subjects and gait instances are presented in Table 3. The average compressive strain without the ICS intervention at ES was −722 ± 155 μE while the value for high containment of the ischial tuberosity was −105 ± 42 μE, showing that the mechanical stimulus increased around seven times in the absence of ICS. The average compressive strain values for the medium and low containment conditions at ES were 208 ± 44 μE and 253 ± 51 μE, respectively, also significantly lower than those without ICS.

**TABLE 3:** Mean compressive strain values during gait instances across 10 subjects.

	Amount of bony pelvic containment											
	0 mm—High			3 mm—Medium			5 mm—Low			without ICS		
	ES	MS	TS	ES	MS	TS	ES	MS	TS	ES	MS	TS
CT01	−184	−65	−88	−248	−181	−215	−309	−192	−280	−629	−402	−598
CT02	−55	−92	−56	−157	−158	−141	−196	−224	−194	−669	−647	−781
CT03	−100	−64	−212	−253	−218	−328	−294	−228	−380	−707	−411	−952
CT04	−44	−97	−169	−127	−166	−233	−188	−224	−284	−576	−579	−594
CT05	−137	−141	−97	−240	−259	−144	−289	−332	−144	−797	−798	−996
CT06	−124	−73	−118	−174	−169	−190	−197	−237	−242	−1060	−878	−1190
CT07	−130	−134	−90	−230	−245	−178	−314	−309	−238	−898	−792	−1020
CT08	−103	−116	−151	−195	−235	−227	−195	−235	−227	−618	−586	−744
CT09	−47	−82	−67	−184	−82	−193	−250	−144	−267	−761	−666	−774
CT10	−126	−49	−99	−267	−185	−215	−301	−235	−309	−509	−417	−597
Mean	−105	−91	−115	−208	−190	−206	−253	−236	−257	−722	−618	−825
SD	42	29	46	44	49	50	51	50	61	155	163	196

*Note:* Scenarios where the remodelling threshold (300 μE) was exceeded are highlighted (in orange).

Abbreviations: ES, early stance; ICS, ischial containment socket; MS, mid stance; SD, standard deviation; TS, terminal stance.

**FIGURE 6:**
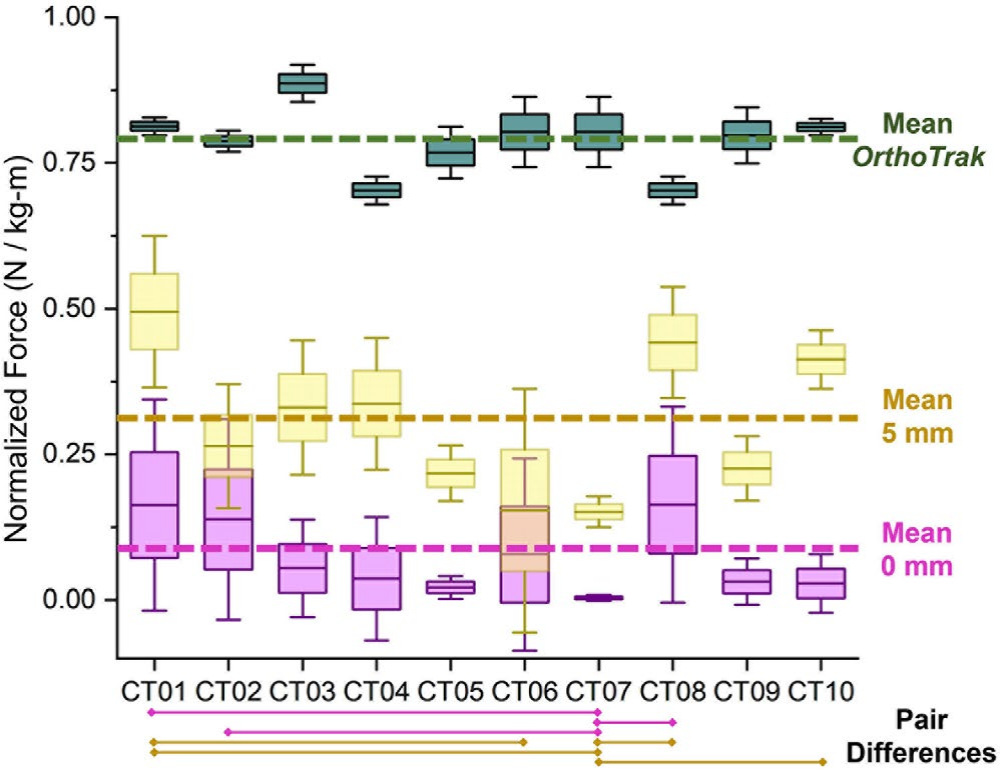
Comparison of OrthoTrak vertical normalised hip joint forces during early stance (green boxes) and FE computed force for two pelvic bony containment amounts: high, 0 mm separation (purple boxes) and low, 5 mm separation (yellow boxes). Dashed lines indicate the mean normalised hip joint forces for each case. Subjects' mean paired differences during 0 mm separation are indicated by solid purple lines, similarly solid yellow lines indicate the paired differences at 5 mm separation.

FE normalised hip joint forces with ICS were considerably lower in comparison to OrthoTrak estimated mean values. The percentage of mean computed values with ICS in comparison to those obtained with the motion analysis software during ES were: 8.6% at 0 mm, 25.5% at 3 mm, and 36.2% at 5 mm. Similarly, for MS the values were: 7% at 0 mm, 24.6% at 3 mm, and 35.5% at 5 mm, and during TS values were: 4.5% at 0 mm, 20.9% at 3 mm, and 29.6% at 5 mm. Post hoc ANOVA tests were used to assess whether the socket created a similar normalised force at the hip joint between subject pairs. The analysis indicated that the effect of the socket is similar between subject pairs despite their different heights, weights, and bone structural geometry, that is, if there is inter‐subject similarity in the results of Figure 6. No significant difference existed across mean values at the 0.05 sensitivity level in the resulting 405 pairings (combination of pairs among ten subjects for three gait instances and three pelvic loading conditions) except in twenty‐three pairs as described in Table 4.

**TABLE 4:** Subjects' hip force pairings with statistical differences during gait instances due to ICS (no statistical difference was found for the remaining 382 pairings for *p* = 0.05).

Gait instance	Pelvic containment	Pair differences	*p*
Early stance	0 mm	CT01 vs. CT07	0.0141
		CT02 vs. CT07	0.0157
		CT07 vs. CT08	0.0065
	3 mm	CT01 vs. CT05	0.0351
		CT01 vs. CT06	0.0041
		CT01 vs. CT07	0.0046
		CT06 vs. CT08	0.0157
		CT07 vs. CT08	0.0175
	5 mm	CT01 vs. CT06	0.0101
		CT01 vs. CT07	0.0036
		CT07 vs. CT08	0.0267
		CT07 vs. CT10	0.0237
Mid stance	3 mm	CT07 vs. CT10	0.0283
	5 mm	CT06 vs. CT10	0.0126
		CT07 vs. CT10	0.007
Terminal stance	0 mm	CT05 vs. CT06	0.0453
		CT06 vs. CT07	0.0065
	3 mm	CT03 vs. CT04	0.0444
		CT03 vs. CT07	0.0091
		CT07 vs. CT10	0.0351
	5 mm	CT03 vs. CT06	0.0065
		CT06 vs. CT10	0.0023
		CT07 vs. CT10	0.0463

## 4 Discussion

The central aim of this study was to assess the mechanical environment and altered loading created by an ICS and its influence on the residual femur as a possible explanation for the reported BMD loss in persons with TFA. The results suggest that the presence of the socket lowers the compressive strain environment in the residual femur throughout the gait cycle as a result of the load transmission interaction between the ischial tuberosity and the ICS.

Our results are consistent with radiographical evidence of low BMD levels reported after a TFA [3, 8, 33]. Average compressive strain values during gait, obtained across all subjects wearing an ICS, showed a significant decrease related to the amount of pelvic bony containment. Moreover, the observed reduction in compressive strain values was not dependent on the subject, position of the prosthetic leg (i.e., gait adaptation), bone geometry, or ground reaction forces experienced during the selected gait instances, suggesting that the dominant contributor was the mechanical effect of the ICS and the clinician's ability to achieve pelvic containment. Mirror models replicating the gait position of those with TFA but without an ICS were capable of reaching strain values at levels required for bone remodelling [34], as reported in Table 3 and seen in Figure 5. This also indicates that, in the absence of ICS and its pelvic support, the mechanical environment reflects a healthy state throughout the stance phase.

Results from our FE models depict a residual femur in an impoverished loading environment, suggesting that while the ICS design for transmitting ground forces through the socket and into the pelvis is successful, it comes at the cost of significantly reducing strain in the femur that could prevent bone loss. This localised reduction of load transmission to the acetabular region has not been previously considered as a possible reason for BMD loss, though the creation of a mechanical stop between the ischial tuberosity and the ICS, which we found transmits 65%–95% of expected hip joint forces, is clinically expected in an ICS [19]. Hence, this two‐end force isolation scenario in the residual femur results in its removal from the kinetic chain and into a flotation state, placing portions of it into the disuse window as described in the Mechanostat theory for bone remodelling [21, 34, 35, 36]. On the whole, our simulations show that the standard‐of‐care prosthetic socket for TFA [17, 18] transforms the mechanical environment in the residual femur because of its pelvic reliance. In contrast, recent studies of individuals with TFA who have opted for a surgically bone‐anchored prosthesis have shown BMD improvement in the residual femur [37, 38]. Given that residual femur loading via pelvic containment affects BMD, additional research into prosthetic designs such as subischial sockets, which eliminate dependence on pelvic support without compromising comfort or function [26, 27] is needed to assess whether they modify the long‐observed demineralization pattern in individuals with TFA.

## 5 Limitations

There are a few study limitations to consider when interpreting the results of these models. First, the models were constructed using CT scans from healthy subjects that were computationally modified to match the residual limb length and gait analysis data of individuals with TFA. Variables known to affect bone material properties such as sex, race, age, and time from amputation were not considered. While skeletal geometries and gait parameters are not from the same subjects, the set‐up is considered sufficiently representative to simulate the mechanical environment created by the ICS.

Second, the models do not consider the contribution of muscular contraction on bone contact forces given that muscle insertion points would be dependent on surgical procedures and therefore vary across individuals. As such, joint contact forces and skeletal compressive strain may be underestimated [39]. Similarly, the analysis of gait instances omits the contributions from motion.

Third, skeletal mechanical properties were defined as isotropic and homogeneous, consistent with previous reports [40], but not reflective of the true heterogeneity of bone properties, and this may have some influence on strain magnitudes.

## 6 Conclusions

FE models simulating the loading environment for an individual with TFA walking in an ICS suggest that the pelvic support provided by the socket design has an influence on the mechanical environment of the residual femur. By limiting forces to the distal residual femur, the ICS renders the residual femur in a partial flotation state, thereby attenuating proximal forces in the acetabular region and suppressing the mechanical stimulus. In summary, the ICS likely contributes to the BMD loss reported in persons with TFA. Prosthetic sockets designed without pelvic support may help improve the mechanical environment necessary for BMD maintenance.

## Author Contributions

J.L.Z.‐R., M.J.M., and P.P. conceived the presented idea and developed the theory at their corresponding institutions. J.L.Z.‐R. performed the computations and statistical analysis at the University of Edinburgh. P.P. verified the analytical methods at the University of Edinburgh. M.J.M. and S.F. provided clinical data from Northwestern University and clinical insights. All authors discussed the results and contributed to the final manuscript.

## Ethics Statement

Study participants were not involved in the design, conduct, interpretation, or translation of the current research. Data from cited study DOI https://doi.org/10.1002/jor.22040 (https://mrl.sci.utah.edu/software/hip‐image‐data) have been made available by the authors of the cited study and had institutional review board approval and informed consent from test subjects. Gait data from cited randomised crossover trial (DOI https://doi.org/10.1016/j.apmr.2021.05.016) had institutional review board approval and informed consent from test subjects.

## Conflicts of Interest

The authors declare no conflicts of interest.

## Data Availability

CT scan data that support the findings of this study are openly available from Weiss Biomechanics Lab at https://weisslabutah.org/. Gait data that support the findings of this study are available from the corresponding author upon reasonable request.
